# A revised key to the Neotropical cleptoparasitic anthidiine genera (Hymenoptera, Megachilinae) with notes and description of the male of *Rhynostelis* Moure & Urban

**DOI:** 10.3897/zookeys.249.4030

**Published:** 2012-12-07

**Authors:** Danúncia Urban, Daniele Regina Parizotto

**Affiliations:** 1 Laboratório de Biologia Comparada de Hymenoptera, Departamento de Zoologia, Universidade Federal do Paraná, Caixa Postal 19020, 81531-980, Curitiba, Paraná, Brazil

**Keywords:** Anthidiini, Brazil, cleptoparasitism, host, taxonomy

## Abstract

*Rhynostelis* Moure & Urban is a monotypic cleptoparasitic neotropical anthidiine genus currently known from two females. Herein, we describe and illustrate for the first time the male and its genitalia and it is confirmed that *Rhynostelis* parasitizes nests of *Eufriesea*. An identification key to the genera of cleptoparasitic anthidiine from the Neotropical region is also presented.

## Introduction

Cleptoparasitism is a common behavior among species of Anthidiini and presumably evolved multiple times within the tribe (Gonzalez et al. 2012). This condition is currently known in eleven genera worldwide, seven of which occur in the neotropical region, *Austrostelis* Michener & Griswold, 1994; *Dolichostelis* Parker & Bohart, 1979; *Heterostelis* Timberlake, 1941; *Hoplostelis* Dominique, 1898; *Rhynostelis* Moure & Urban, 1995; *Stelis* Panzer, 1806; and the recently described *Melostelis* Urban, 2011, which is known from a single female from the Amazon (Michener 2007, Moure and Urban 2007). In the classification proposed by Michener (2007), *Austrostelis*, *Hoplostelis* and *Stelis* are treated as genera, with remaining taxa treated as subgenera. In this contribution, all taxa are treated at the generic level, according to the classification of Urban and Moure (2007).

Little is known about the biology of these cleptoparasitic bees. In some genera, as *Hoplostelis* and *Dolichostelis*, the female enters the nest of a host and kills its eggs and/or larvae. In other groups, such as *Stelis*, the female lays her eggs on the nest while still open and the young larva has mandibles adapted to kill the host egg or larva (Michener 2007). Cleptoparasitic Anthidiini mostly parasitizes other Anthidiini, but they can also parasite other Megachilinae genera well as Euglossini (Apinae). *Austrostelis* was recorded by Zanella and Ferreira (2005) from a nest of *Epanthidium tigrinum* (Schrottky, 1905); *Hoplostelis* is known to parasitize the orchid bee *Euglossa cordata* (Linnaeus, 1758) (Bennett 1966); *Dolichostelis* is cleptoparasitic in nests of *Megachile* Latreille, 1802, probably of the subgenus *Megachile* (*Chelostomoides*) Robertson, 1901 (Parker and Bohart 1979); *Stelis* parasitizes several genera of Megachilinae: *Anthidiellum* Cockerell, 1904, *Anthidium* Fabricius, 1804, *Ashmeadiella* Cockerell, 1897, *Chelostoma* Latreille, 1809, *Heriades* Spinola, 1808, *Hoplitis* Klug, 1807, *Megachile* Latreille, 1802 and *Osmia* Panzer, 1806 (Michener 2007); *Heterostelis* is known to parasitize species of *Trachusa* Panzer, 1804 (Thorp 1966 and Michener 2007).

*Rhynostelis* consists of a single species, *Rhynostelis multiplicata* (Smith, 1879), which is known from two female specimens collected on the State of Amazonas, Brazil. Herein, we present an updated diagnosis of *Rhynostelis*, describe and illustrate the male of *Rhynostelis multiplicata* and, based on two specimens collected from a trap-nest, we record the orchid bee *Eufriesea laniventris* (Ducke, 1902) as its host. A key to all neotropical cleptoparasitic anthidiine genera is also provide.

## Material and methods

Morphological terminology follows Michener (2007) and that for mandibles Michener and Fraser (1978). Measurements are given in millimeters and were taken using an ocular micrometer on a stereoscopic microscope Leica MZ125. Total length was measured in lateral view, from the head to the apex of metasoma; length of forewing was measured at the anterior margin, from the costal sclerite to the wing apex. The illustrations were obtained with a Leica DFC 500 digital camera attached to the stereoscopic microscope Leica MZ 16 and combined with the software AUTO-MONTAGE PRO. All material used in this study is deposited in the Coleção Entomológica Pe. Jesus Santiago Moure, Universidade Federal do Paraná, Curitiba, Brazil (DZUP).

## Taxonomy

### Rhynostelis

Moure & Urban, 1995

http://species-id.net/wiki/Rhynostelis

Figs 1–7 15, 17

Rhynostelis Moure & Urban, 1995: 297.
Hoplostelis (*Rhynostelis*) Michener, 2007: 518.

#### Male description.

**Figures 1–7.**
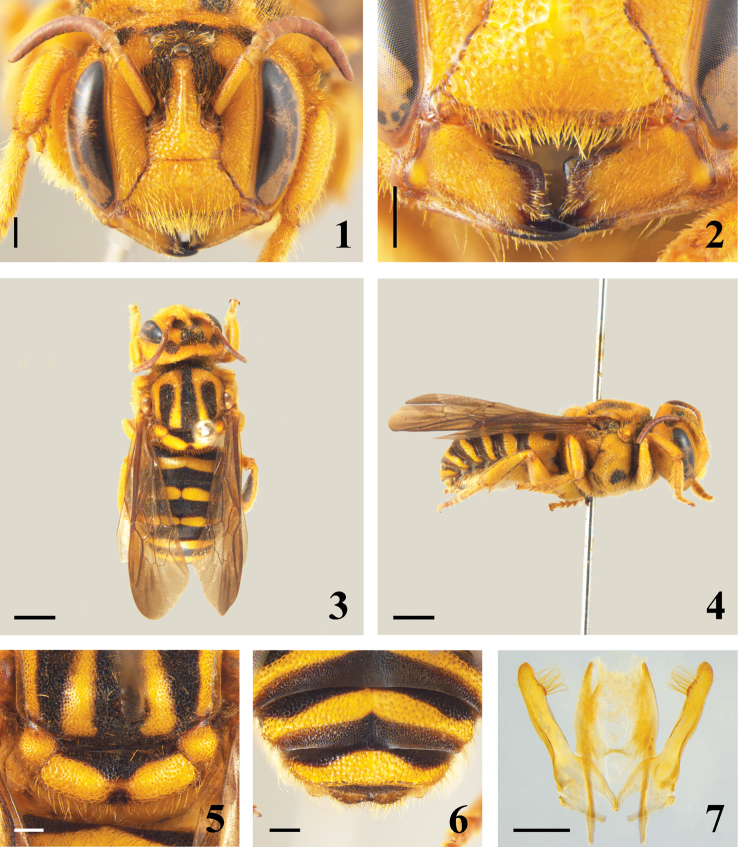
Male of *Rhynostelis multiplicata*. **1**head in frontal view **2** detail of mandibles **3** dorsal view **4** lateral view **5** detail of scutellum **6** apex of metasoma **7** male genitalia in dorsal view. Scale line = 0.5 mm (Figures **1–2, 5–7**). Scale line = 2.0 mm (Figures **3–4**).

**Diagnosis**. Mandibles with only apical tooth elongated, upper distal angle rounded (Fig. 2); clypeus protuberant, with a basal tubercle at middle; supraclypeal area protuberant (Fig. 1); scutum and scutellum bigibbous; axillae and dorso-ventral area of mesepisternum gibbous (Figs 3, 5); distal tergum short and sinuous at middle (Fig. 6); sternum sixth with laminar rounded, lateral projections.

#### Description.

Mandibles with distal margin almost straight, only with apical tooth elongated; upper distal angle slightly marked, condylar carina elevated and strongly laminated, slightly wider at base (Fig. 2). Clypeus protuberant with median basal tubercle elongated, extended in low irregular carina in basal half, apex depressed without projections, not exceeding labrum, covered by many long hairs. Labrum weakly bilobed near clypeal base. Supraclypeal area with wide, somewhat flat median carina; frons with long, well marked carina. Juxtantennal carina laminated, ventrally short, not arising at base of antennal sockets and extending upward (Fig. 1). Scutum and scutellum bigibbous; axillae gibbous; base of propodeum with irregular foveae (Fig. 3, 5). Omaulus lamellate, almost extending ventrally; mesepisternum with gibbous area near mesocoxal cavity. Fore and middle tibiae with midapical spine on outer surface (Fig. 15); arolia present. Fifth and sixth terga with transverse, median low carina; sixth tergum with apical projection at middle; distal tergum slightly emarginated at apex (Fig. 6). Second sternum enlarged, with apex weakly emarginated at middle, with long pilosity at apex; third to fifth sterna with dense apical pilosity, laterally with longer and curved hairs; sixth sternum with distal margin almost straight, with large, angled, laminar, lateral projections subapically.

**Genitalia.** Gonostylus slightly longer than penis valves; apical half with dense and long pilosity on inner margin; apex rounded, laterally slightly convex. Gonobase incomplete dorsally, only visible laterally (Fig. 7).

#### Comments.

Moure and Urban (1995) mentioned that the base of the propodeum in *Rhynostelis* lacks foveae. Michener (2007), also pointed out this feature as one of the characteristics that separates *Rhynostelis* from *Hoplostelis*. However, a re-examination of *Rhynostelis* specimens revealed the presence of irregular fovea in the base of its propodeum, which are difficult to see due the shiny yellow integument. Michener (2007) considered *Rhynostelis* as a subgenus of *Hoplostelis* and in his key, the male agrees with the female by the characters he listed, except for the absence of foveae in the base of propodeum, as commented above. The male of *Hoplostelis* further differs from *Rhynostelis* by the following combination of characters: three strong mandibular teeth and two basal depressions near the articulation of the head; omaulus carinate only in the dorsal one-half or one-third; sixth sternum with narrow lateral projections and without gibbous mesosoma.

### Rhynostelis multiplicata

(Smith, 1879)

http://species-id.net/wiki/Rhynostelis_multiplicata

Figs 1–7 15, 17

Anthidium multiplicatum Smith 1879: 87.
Rhynostelis multiplicata ; Moure and Urban 1995: 298.
Hoplostelis (Rhynostelis) multiplicata ; Michener 2007: 518.

#### Diagnosis.

Integument black, with wide yellow areas in both sexes. Scutum with a large, reverse U-shaped macula; scutellum almost totally yellow; axillae yellow and all terga with yellow bands (Figs 3, 4). Male and female with protuberant clypeus.

#### Male description.

Approximate body length 13.29; forewing length 10.45; head width 4.02; head length 3.67; eye length 2.55. Head integument yellow except: distal margin of mandible and apical tooth black; labrum blackened; frons black, bands above superior margin of antennal sockets extending to vertex, including ocelli and finely attached with black spot above compound eyes. Antennae with ventral face of pedicel darkish yellow, remaining segments light brown; dorsal face with scape and pedicel light brown and flagellum amber (Figs 1, 2). Pronotal lobe yellow; scutum black with large reverse U-shaped yellow maculae; scutellum with on yellow gibbous area joined medially by fine yellow band; axillae yellow; metanotum brown and propodeum yellow. Mesepisternum and metepisternum yellow; mesepisternum with discal black spot, metepisternum with ventral area black. Tegula amber and wing membrane brown. Legs almost totally yellow; middle tibiae with internal darkish area and hind tibiae with large internal darkish area (Figs 3, 4, 5). Terga black; basal tergum with yellow band slightly angled at middle; second to fourth with large yellow band, slightly narrower and slightly interrupted medially; fifth tergum with yellow band emarginated at middle on posterior margin; sixth tergum with yellow band, wider medially; distal tergum with subapical yellow band and blackish margin (Fig. 6). Two basal sterna yellow, with large translucent margin; third to fifth yellow with black infumated area on apical half; sixth sternum with large median black apical spot.

Pubescence. Light yellow with predominantly short hairs (less than ocellus diameter). Pilosity longer and denser among ocelli, above antennal sockets and clypeus apex. Hairs of mesepisternum little longer than mesoscutum, curved on scutellum and propodeum. Fore leg with coxa and trochanter covered by dense pilosity. Third to fifth sterna with apical dense and long slight curved hairs.

Sculpturing. Head finely punctate with sparser punctures on clypeal protuberance and at supraclypeal area. Mandibles with punctures smaller than that of head. Mesoscutum with integument microreticulated, punctures deeper than head. Gibbous area of mesepisternum with larger and sparser punctures; distance between punctures at least half width a puncture diameter. Punctures of terga fine and shallow; punctures on yellow bands larger and sparser than those on black areas.

Material examined. BRAZIL, *Pará*, Belém: one male, “IPEAN [Instituto de Pesquisas e Experimentação Agropecuária do Norte, Belém] / 105-2 / EU” (DZUP); and one female “IPEAN / 105-1/ EU” (DZUP). *Amazonas*, Manaus: one female “Proj. DBFF.WWF/ Manaus-AM/ Brasil 04/11/89 / M. B. V. Garcia (DZUP).

Remarks on the female from Pará. Approximate body length 16.13; forewing length 12.38; head width 4.90; length head 4.16, eye length 3.18. Integument predominantly yellow. The female differs from the male as follows: black maculae on vertex and frons larger than in male. All terga with yellow bands; basal tergum with complete band; second and third terga with bands slightly interrupted at middle; fourth tergum with band interrupted anteriorly only at middle, fifth tergum with band interrupted only posteriorly at middle; sixth tergum with subapical band and blackish margin. All sterna yellow; distal sternum with medioapical spot and black margin. The female collected in Manaus is a little smaller than the female from Belém and the integument of the head is darker and with irregular macula. Such differences in color might be caused by some chemical product used during collection or preservation.

Host records. Urban and Moure (1995) commented that one female of *Rhynostelis multiplicata* emerged from a test-tube placed on a termite nest where an orchid bee was previously seen (possible *Eufrisea pulchra* (Smith, 1854)). However, the record of the host was never confirmed. The specimens from Belém studied herein emerged from one nest of *Eufriesea laniventris* (Ducke, 1902), (DZUP), according the identification of Dr. Gabriel A. R. Melo. Thus, the cleptoparasitism of *Rhynostelis multiplicata* on orchid bees of the genus *Eufriesea* is confirmed.

**Figures 8–19.**
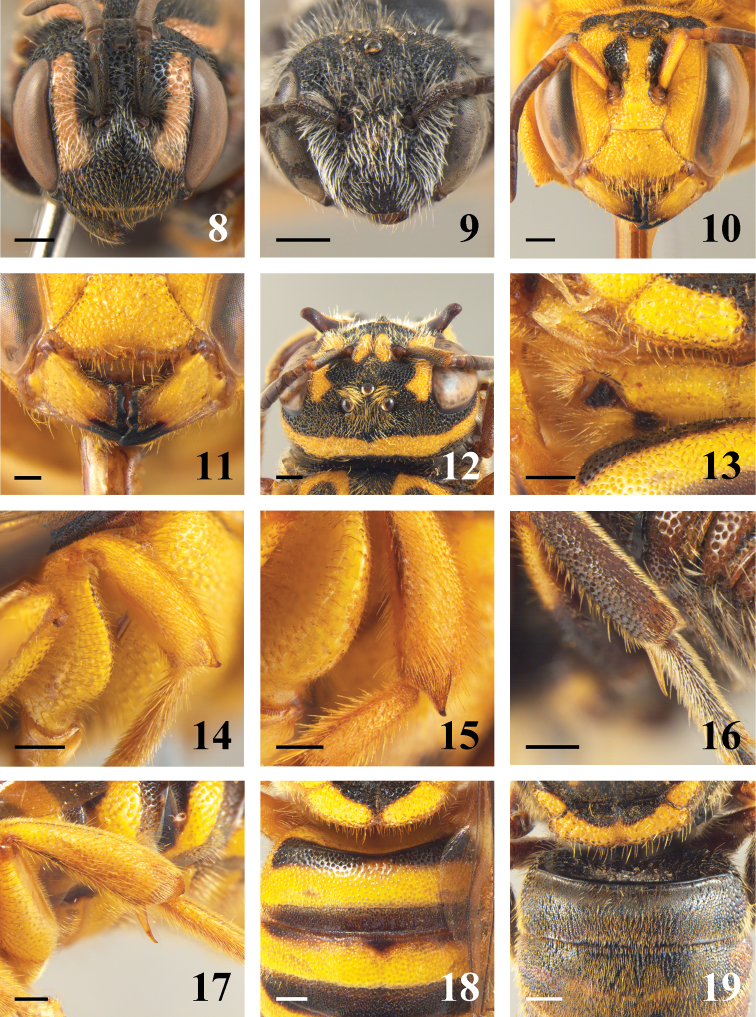
**8–10** head in frontal view of female. **8** *Dolichostelis louisae* **9** *Stelis lateralis* **10** *Melostelis amazonensis* **11** details of mandibles of *Melostelis amazonensis* **12** vertex of *Hoplostelis bilineolata* **13** fovea of propodeum of *Melostelis amazonensis* **14–15** middle tibia of female **14** *Melostelis amazonensis* **15** *Rhynostelis multiplicata* **16–17** hind tibia of female **16** *Hoplostelis bilineolata* **17** *Rhynostelis multiplicata* **18–19** details of first tergum of metasoma of female **18** *Melostelis amazonensis* **19** *Hoplostelis bilineolata*. Scale line = 0.5 mm (Figures **8–19**).

##### Identification key to the neotropical cleptoparasitic genera of Anthidiini

**Table:** 

1	Juxtantennal carina absent; middle tibia with two widely separated apical spines	2
–	Juxtantennal carina present; middle tibia with one apical spine, or subapical carina	4
2(1)	Hind tibia with subapical spine near posterior margin, sometimes completely hidden by dense hairs; hind basitarsus with carina along inner dorsal angle, separated from margin by a longitudinal depression	*Heterostelis*
–	Hind tibia without subapical spine; hind basitarsus without carina	3
3(1)	Anterior surface of mesepisternum with punctures sparser than on lateral surface; omaulus carinate; interalveolar area short and protuberant (Fig. 8). Male: third sternum with pair of translucent lobes; forth sternum with two median projections	*Dolichostelis*
–	Mesepisternum uniformly punctate; omaular carina absent; interalveolar area flat (Fig. 9). Male: third sternum not modified and fourth sternum with a setose medioapical projection	*Stelis*
4(1)	Body relatively elongated; small-sized species. Integument coarsely punctate; pilosity between ocelli not differentiated from remaining areas of head	*Austrostelis*
–	Body robust, metasoma almost globose, large species (Fig. 3, 4). Integument with fine to moderate-sized punctures; pilosity among ocelli longer than in remaining areas of head (Fig. 12)	5
5(4)	Scutellum laterally with laminate distal margin, with punctures at least twice as larger as those on scutum; hind tibia without flat apical projection on outer surface (Fig. 16); basal tergum with transverse carina (Fig. 19)	*Hoplostelis*
–	Scutellum without laminate projection, with punctures about as large as those on scutum; hind tibia with flat apical projection on outer surface (more developed in females) (Fig. 17); basal tergum without carina (Fig. 18)	6
6(5)	Frons not carinate; clypeus without basal tubercle and with two apical projections (Fig. 10). Mesosoma not gibbous; postspiracular fovea drop-shaped, microreticulated, coarsely punctuate and with irregular alveoli (Fig. 13). Female: mandible without bifurcated condylar carina and without protuberance near anterior articulation (Fig. 11); fore and middle tibiae with apical spine as long as half width of median ocellus diameter (Fig. 14)	*Melostelis*
–	Frons carinate; clypeus with basomedian tubercle and without apical projections (Fig. 1). Scutum and scutellum bigibbous (Fig. 5); postspiracular fovea rectangular and with regular alveoli. Female: mandible with bifurcated condylar carina elevated and with protuberance near anterior articulation; fore and middle tibiae with apical spine as long as a diameter of median ocellus (Fig. 15)	*Rhynostelis*

## Supplementary Material

XML Treatment for Rhynostelis

XML Treatment for Rhynostelis multiplicata
